# Modulation of neuronal excitability by binge alcohol drinking

**DOI:** 10.3389/fnmol.2023.1098211

**Published:** 2023-02-14

**Authors:** Pablo Gimenez-Gomez, Timmy Le, Gilles E. Martin

**Affiliations:** ^1^Department of Neurobiology, University of Massachusetts Chan Medical School, Worcester, MA, United States; ^2^The Brudnick Neuropsychiatric Research Institute, Worcester, MA, United States; ^3^Graduate Program in Neuroscience, Morningside Graduate School of Biomedical Sciences, UMass Chan Medical School, Worcester, MA, United States

**Keywords:** alcohol, binge alcohol drinking, neuroadaptation, neuronal excitability, ion channel

## Abstract

Drug use poses a serious threat to health systems throughout the world. The number of consumers rises every year being alcohol the drug of abuse most consumed causing 3 million deaths (5.3% of all deaths) worldwide and 132.6 million disability-adjusted life years. In this review, we present an up-to-date summary about what is known regarding the global impact of binge alcohol drinking on brains and how it affects the development of cognitive functions, as well as the various preclinical models used to probe its effects on the neurobiology of the brain. This will be followed by a detailed report on the state of our current knowledge of the molecular and cellular mechanisms underlying the effects of binge drinking on neuronal excitability and synaptic plasticity, with an emphasis on brain regions of the meso-cortico limbic neurocircuitry.

## 1. Introduction

Traces of alcohol, an organic compound that results from the fermentation of grain, fruit juice and honey, have been identified in early 7,000 BC settlements in China (McGovern et al., 2004) and in jars from the Middle East and Egyptian settlements dating back to 3,000 BC (Cavalieri et al., 2003; Gately, 2008). These archeological findings arguably place alcohol as one of the first, if not the first, drug used by humans. Today, it is the most widely used and abused legal drug, becoming a major global health problem as the United Nations Office on Drugs and Crime admits. Worldwide, 57% of the global population aged 15 years and over consumed alcohol in the previous 12 months producing 3 million deaths (5.3% of all deaths) worldwide and 132.6 million disability-adjusted life years. In the U.S. alone, excessive alcohol consumption is responsible for 13% and 20% of total deaths among adults 20 to 64 and 20 to 49 years old, respectively (Esser et al., 2022). Alcohol is consumed in a variety of methods, from light social drinking to more abusive forms. Early enquiries focused primarily on the development of tolerance and dependence resulting from prolonged and repeated heavy drinking. Over the past decade, binge alcohol drinking has received increased attention in part due to the recognition that it is the preferred mode of consumption of adolescents and young adults. As school surveys show alcohol use starts before the age of 15 and the prevalence of alcohol use can be in the range of 50%–70% with no remarkable differences between sexes (Substance Abuse and Mental Health Services Administration, 2019). Moreover, the prevalence of heavy episodic drinking using binge alcohol drinking is 18.2% with a peak in young adults, an age group particularly susceptible to the drug effects owing to the biological vulnerability of their still developing brains (Kwan et al., 2020). In the present review we tried to discuss all the relevant bibliography for the topic discussed. In order to do so we used Pubmed as a main resource for publications using keywords such as “alcohol,” “EtOH,” “Ethanol,” “Drinking in the dark” or the name of other relevant models along with keywords related to each topic discussed (Neuronal excitability, potassium channels, sodium, channels, ion channels, neuronal firing, synaptic transmission, plasticity, glutamate, GABA, glutamate receptors, gaba receptors, AMPA, NMDA or synaptic gating).

### 1.1. Consequences of binge alcohol drinking on behavior

As originally described by Tomsovic (1974), binge alcohol drinking is defined as repeated periods of heavy drinking, followed by periods of abstinence. Although the number of drinks consumed during a 2-h period was initially considered as the main marker, a recent attempt to standardize studies led to an updated definition stipulating that binge drinking must elevate blood alcohol levels to 80 mg/dl or above. This typically corresponds to consuming five or more drinks (male), or four or more drinks (female), in approximately 2 h (NIAAA, 2004). Such drinking pattern has been primarily studied in young adults (e.g., college students) as they represent by far the largest group affected by this behavior (Naimi et al., 2003), although this drinking pattern is also increasingly common in adolescents as young as 12 (Sun et al., 2008; SAMHSA, 2014). Furthermore, this pattern of drinking may be exacerbated in adolescents and young adults during the 2020 COVID-19 lockdown as a means to cope with social isolation (Skrzynski and Creswell, 2020).

Regarding the brain areas affected by binge alcohol drinking, there is strong cumulative evidence that the frontal lobes, which mature later compared to the rest of the brain (Giedd et al., 1999) and play a major role in controlling inhibitory responses, are particularly susceptible to this pattern of consumption. Accordingly, just one episode of binge drinking during 3 months produces thinner and lower volumes of the prefrontal cortex (PFC) and cerebellar regions, and attenuated white matter development (Cservenka and Brumback, 2017). These changes translate into alterations in a number of cognitive tasks such as self-control, working memory, decision making and social and emotional processing. Thus, Scaife and Duka (2009) showed specific impairments in the dorsolateral PFC of females, and the temporal lobes of males and females in binge drinkers compared to non-drinkers. Not only does heavy binge alcohol drinking potentially lead to destructive behaviors such as suicide, drunk driving (Wechsler et al., 1994), and cognitive deficits (Duka et al., 2004), but it is also recognized as being the precursor to long-term alcohol-related problems like sleep disorders, stroke, and social anxiety (Townshend and Duka, 2002; Weissenborn and Duka, 2003; Hartley et al., 2004). Importantly, binge drinkers, contrary to light drinkers, typically display a stronger response to early euphoric effects but are less sensitive to the sedative effects of alcohol, indicative of a predisposition for the development of alcohol addiction (Schuckit, 1994; Schuckit et al., 2008). While the PFC appears particularly vulnerable (Moorman, 2018), probing the effects of binge drinking showed ethanol widespread reach to other brain regions, as we will describe below, including those associated with the neurocircuitry of drug addiction.

Over the past two decades, spurred by the need to work with animals whose blood alcohol levels could be rapidly raised to 80 mg/dL or higher, a level that is generally considered to be intoxicating and representative of excessive drinking (Bell et al., 2006), a number of models were developed, ranging from alcohol consumption without access to water, to gavage, two-bottle choice, and alcohol liquid diet (Crabbe et al., 2011). Two additional protocols seemed to have gathered a general agreement regarding their usefulness. Indeed, most of the data discussed here have mostly been obtained with these two models, the Drinking-in-the-dark (DID) and the chronic intermittent alcohol vapor exposure (CIE) paradigms. DID protocol, originally described by Rhodes et al. (2005), consists in substituting the free access to water to free access to EtOH during 2 h the first 3 days and 4 h during the last day. Ethanol is offered at a concentration of 20% in tap water and the mice drink voluntarily (Thiele and Navarro, 2014). CIE is an EtOH dependence and relapse drinking model described by Becker and Lopez which *via* repeated cycles of chronic intermittent exposure to EtOH vapors in inhalation chambers and periods of withdrawal generate an escalation in voluntary EtOH consumption simulating the transition to EtOH dependence in mice (Becker and Lopez, 2004) and rats (Gilpin et al., 2008). The chief advantages of the DID model are its simplicity, its short duration, as well as its ability to quickly drive blood alcohol concentrations to levels associated with robust intoxication (Ryabinin et al., 2003; Rhodes et al., 2005; Sharpe et al., 2005). Although more complex and costly in its implementation, CIE also promotes a rapid escalation and large EtOH intake (O’Dell et al., 2004). The chief advantage of the DID strategy is that mice can reach intoxication levels at a faster rate relative to that of the CIE model (the mice only need 4 days of consumption using DID protocol; Thiele and Navarro, 2014). Furthermore, the DID is a cost-effective strategy do not require the delivery of vapors or any type of injections (e.g., CIE protocols usually requires the use of Pyrazole and EtOH i.p). On the other hand, the CIE model, unlike DID, produces symptoms in mice compatible with dependence (Becker and Lopez, 2004).

## 2. Regulation of neuronal excitability by binge drinking

In contrast to its deleterious effects on the liver, heart, blood, and pancreas that typically appear following prolonged and repeated heavy consumption (Rehm et al., 2009; Mostofsky et al., 2016), alcohol affects brain functions as early as the first experience and at lower concentrations (Mukherjee, 2013). With the principal consequences of binge drinking being behavioral alterations, it is imperative to identify the origins of these effects. Considering that the primary function of neurons is to transport information from one part of the brain to another, early research sought to identify the cellular mechanisms underlying alcohol effects on neuronal excitability. The ability of nerve cells to fire action potentials, the electrical signals that support cell-to-cell communication, results from a subtle balance between neuronal intrinsic and extrinsic (i.e., synaptic) homeostatic states (Franklin et al., 1992; Turrigiano et al., 1994). While intrinsic excitability is controlled by a variety of sodium, potassium and calcium channels, synaptic transmission results from the interplay between the release of neurotransmitters, the availability of their endogenous receptors and their biophysical and pharmacological properties defined by their subunit composition (Citri and Malenka, 2008). Additionally, these features are tightly controlled by a number of neuromodulators such as Neuropeptide Y (van den Pol, 2012) Corticotropin Releasing Factor (Joshi et al., 2020) or dopamine (Tritsch and Sabatini, 2012). We will review the state of our current knowledge about the various mechanisms that binge drinking employs to alter behaviors through its effects on neuronal excitability. Although, for clarity, we have classically divided the molecular targets of binge drinking in broad categories, it should not be construed that they are strictly independent and compartmentalized. Instead, their activation is carefully orchestrated as exemplified with voltage-gated sodium and calcium channels that amplify synaptic potentials in dendrites of hippocampal CA1 pyramidal neurons (Magee and Johnston, 1995) while hyperpolarization-activated cyclic nucleotide-gated cation channels (HCN) inhibit EPSPs the same neurons (George et al., 2009).

## 3. Binge drinking regulation of ion channel properties and neuronal excitability

### 3.1. Potassium channels

In addition to shaping neuronal excitability, voltage-gated K^+^ channels regulate synaptic transmission and plasticity as they are recruited during depolarization induced by synaptic events (Frick et al., 2004; Truchet et al., 2012).

Therefore, changes in their expression and biophysical properties in the presence of alcohol may directly affect the excitability of neurons and their ability to integrate and process synaptic events. As pharmacological tools and electrophysiological techniques improved, so did the isolation of many ion channels that had previously been inaccessible. However, given the staggering diversity of these channels, compounded by the existence of a number of splice variants for some of these genes, and the sheer difficulty for some to be fully isolated from other ionic currents, only a few have been thoroughly characterized.

It is worth noting that the sensitivity of K^+^ channels to physiologically relevant concentrations of acute EtOH (i.e., ≤50 mM) is generally weak. While, in Xenopus oocytes, Kv4 (mShall) and Kv1 (Shaker) channels are insensitive to ethanol, Kv3 (Shaw2) potassium currents are inhibited at only very high concentrations (i.e., ≥100 mM; Covarrubias and Rubin, 1993), confirming an earlier study where none of the 10 different voltage-gated K+ channels expressed in oocytes were sensitive to EtOH (Anantharam et al., 1992). Similarly, in invertebrates, A-currents (I_A_) are marginally inhibited by very high EtOH concentrations (Alekseev et al., 1997), and the activity of the G-protein-activated inwardly rectifying potassium channels (GIRKs), in both homomeric and heteromeric forms, is significantly enhanced only at highly intoxicating concentrations of alcohol, an effect believed to depend on the interactions of EtOH with a short sequence of 43 amino acids in the carboxyl terminus (Kobayashi et al., 1999; Lewohl et al., 1999). However, exceptions to this seeming rule exist. One is the calcium- and voltage-sensitive potassium channel (KCNM, mSlo) or BK channel. This channel, that strongly repolarizes membrane potential when activated due to its uniquely large conductance (i.e., ~200 pS and higher), is found in all brain regions of the addiction circuitry. It is potentiated by acute EtOH concentrations as low as 10 mM (Dopico et al., 1996, 1999; Martin et al., 2004). The other is the KCNQ channel which drives the sustained M-current to prevent excessive depolarization by mediating persistent outward K^+^ currents at depolarized potentials. Like the BK channels, KCNQ channels are highly expressed in the cortex, hippocampus and nucleus accumbens (NAc), and are inhibited by low (~10 mM) acute EtOH concentrations in drosophila (Cavaliere et al., 2012).

The molecular site of action of ethanol has been a long-standing question in the field of alcohol research (see Abrahao et al., 2017 for a thorough review). Ethanol was initially thought to solely alter channel properties by interacting with the protein lipid environment, a mechanism that is experimentally supported (Crowley et al., 2003; Yuan et al., 2004; Crowley et al., 2005) albeit at high concentrations of EtOH (Ingólfsson and Andersen, 2011). More recently, an elegant study by Bukiya et al. (2014) identified a distinct pocket as the EtOH-recognition site that is placed between the calcium-sensors and gate of the channel α subunit, supporting a direct interaction between EtOH and the BK channel. Although it is unclear whether a similar interaction exists for other ion channels, this is likely because no dedicated EtOH receptors have so far been identified. This observation followed that of Aryal et al. (2009) who identified the cytoplasmic hydrophobic alcohol-binding pocket in GIRK channels. Recent observations also indicate that ligand-gated ion channels are similarly equipped with an EtOH-binding site (see for review Trudell et al., 2014). These EtOH-protein and EtOH–lipid interactions, which probably coexist, provide alcohol with a wealth of options to modulate channel function. Although a low sensitivity to acute EtOH may seemingly disqualify most K^+^ channels as contributing to the effects of binge drinking, it may not be so. Other factors like channel subunit expression may be equally, if not more important. Indeed, subunit composition is instrumental in regulating the properties of ion channels (Torres et al., 2007), and by extension their influence on neuronal excitability (Brenner et al., 2005). A recent in-depth analysis of the genes encoding various K^+^ channels in the NAc and PFC revealed a significant correlation between K^+^ channel transcripts and voluntary drinking in the naïve BXD strain of mouse (Rinker et al., 2017). This study also reported that the expression of genes encoding a K^+^ delayed rectifier, the A-current, and G protein-gated inwardly rectifying K+ channels (GIRKs) was significantly altered following intermittent alcohol exposure in both the PFC and NAc. Using a two-bottle choice drinking model, McGuier et al. (2016) recently reported that genes that encode Kv7 channels (i.e., KCNQ2/3) are related to alcohol consumption and preference in rodent NAc, confirming previous findings showing differential expression of KCNQ2 in the NAc of mice selectively bred for high alcohol consumption (Metten et al., 2014). Similarly, You et al. (2019) showed that ethanol increases VTA neurons excitability in part by inhibiting KCNK13, a two-pore potassium channel that also contribute to excessive alcohol consumption in binge drinking mice, further underscoring the potential role of voltage-gated potassium channels in mediating alcohol effects on neuronal excitability. These results demonstrate that the lack of sensitivity of some K^+^ channels to acute EtOH is not a reliable criterion to define their contribution to neuronal adaptation in binge drinking animals. Interestingly, ethanol metabolites, such as acetaldehyde and acetic acid, may also contribute to modulating potassium channels function as shown in GH3 cells where acetic acid activates BK channels, leading to membrane hyperpolarization, cessation of Ca2+ oscillations and decrease of growth hormone release (Ghatta et al., 2007; Shaidullov et al., 2021).

### 3.2. Sodium channels

Their importance cannot be overstated given their role not only in helping propagate information to downstream neurons, but also in informing the dendritic arborization on the level of excitability reached by the soma. This retrograde propagation into the dendritic compartment controls the induction of synaptic plasticity by interacting in a precise time-dependent fashion with excitatory postsynaptic potentials in a number of neuronal populations such as pyramidal neurons in the cortex and hippocampus (Caporale and Dan, 2008; Sjostrom et al., 2008) as well as in medium spiny neurons (MSNs) of the dorsal (Fino and Venance, 2010) and ventral striatum (Ji and Martin, 2012; Bosch-Bouju et al., 2016). These channels are formed by the association of a large pore-forming α subunit with accessory proteins or β subunits (Catterall, 2000). Although a single α subunit forms the core of the channel and is functional on its own (Barchi, 1988), each of the various β subunits (β1 to β4) modulates α subunit gating properties in unique ways (Brackenbury and Isom, 2011).

Of particular interest is the β4 subunit, encoded by the Scn4b gene, as it prevents normal inactivation, thus conferring channels the ability to evoke a resurgent current upon repolarization (Grieco et al., 2005; Aman et al., 2009; Bant and Raman, 2010). While all brain regions express one or more variants of the gene encoding the Na^+^ channel α subunit (Goldin et al., 2000; Goldin, 2001), those expressing Scn4b are restricted to discreet brain regions particularly the dorsal striatum and the NAc (Oyama et al., 2006; Miyazaki et al., 2014), where this subunit selectively controls long-term depression (Ji et al., 2017a). Although this channel is insensitive to acute EtOH (Wu and Kendig, 1998), chronic EtOH exposure markedly altered expression of mRNA encoding the Scn4b subunit in alcohol-preferring mice and rats (Mulligan et al., 2006; Tabakoff et al., 2008), and more recently in populations of alcoholics (Farris et al., 2014). While Scn4b subunit may not be directly implicated in regulating alcohol consumption in mice, some evidence suggest that nucleus accumbens sodium leak currents may be responsible for EtOH’s acute hypnotic effects (Blednov et al., 2019) and for alterating alcohol-mediated locomotor sensitization (Wu et al., 2021).

### 3.3. Effects of binge drinking on neuronal firing

In light of these findings, it is reasonable to expect that changes in neuronal intrinsic excitability reflect the high sensitivity of these ion channels to binge alcohol drinking.

No significant difference in the baseline firing rate of VTA DA neurons in slices from alcohol drinking mice compared to EtOH-naïve control mice was reported (Avegno et al., 2016). Doyon et al. (2021) showed that a gradual infusion of ethanol significantly altered VTA DA neurons firing rate in a concentration-dependent manner providing. However, those findings may mask the effects of lower alcohol concentrations. Thus, in the same brain region, Juarez et al. (2017) recently reported higher bursting and firing activity in dopamine neurons, a phenomenon absent in mice consuming higher level of alcohol. In contrast, in the NAc core region, prolonged withdrawal from CIE treatment induces a sharp increase of the inward rectification caused by larger K_ir_ currents (Marty and Spigelman, 2012), these channels are the major determinants of the input resistance and the hyperpolarized resting membrane potential of MSNs during the down-state (Nisenbaum and Wilson, 1995). This effect was associated with a lower input resistance, faster action potentials (APs), and larger fast afterhyperpolarizations (fAHPs) compared to MSNs from control animals. Interestingly, EtOH drinking modulates the expression of mRNAs encoding the K_ir_ channel subunits in the NAc of alcohol-preferring rats (Mulholland et al., 2011). The same study also reported that CIE enhanced I_A_ current amplitudes. Surprisingly, they found no change in the firing rate of MSNs. Using a different consumption protocol, Hopf et al. (2010) showed that a protracted withdrawal following a fixed ratio EtOH self-administration protocol increases the firing of core NAc MSNs, an effect they attributed to the inhibition of SK channels. In the orbitofrontal cortex, chronic intermittent ethanol exposure increased firing in large regular-spiking cells, an effect attributed to a decrease of functional activity of SK channels (Nimitvilai et al., 2016). In the inferior colliculus dorsal cortex, intragastric ethanol also increased intrinsic excitability by lowering the action potential threshold while leaving the resting membrane potential unaffected (Evans et al., 2000). In the central nucleus of the amygdala (CeA), and the ventral bed nucleus of the stria terminalis, binge drinking followed by a 3-day withdrawal period induced a net inhibition and hyperexcitability, respectively (Pleil et al., 2015).

Taken together, these studies underscore the complex interactions of EtOH with neurons of the cortico-limbic circuitry, and highlight the notion that binge drinking impacts neuronal excitability in ways that likely reflect the idiosyncratic expression of a variety of ion channels unique to each brain region and neuronal population. This is well illustrated by the BK channels that controls neuronal repolarization following action potential firing. BK channels present a complex of subunits that include the pore forming α subunit and four regulatory β1-4 subunits, products of four distinct genes. Whereas the BK α subunit is ubiquitously expressed in the brain (Chang et al., 1997), among the β(1–4) subunits, only the β1 and β4 have been reported in the central nervous system (Behrens et al., 2000; Brenner et al., 2000). In rat NAc MSNs, the expression of the β1 and β4 subunits is compartment-specific, with the former expressed in the dendritic arborization while the latter is found mostly in the soma (Martin et al., 2004). To further muddle the picture, their expression patterns is likely reversed in mouse MSNs, illustrating the perils of drawing broad conclusions based on data from one species. Moreover, the sensitivity of BK channels to EtOH is markedly different depending upon the subunit composition of the channels, with the αβ1 subtype being totally insensitive, while the αβ4 subtype is enhanced by the drug (Martin et al., 2004). Although it is unknown whether a comparable differential expression pattern between soma and dendrites apply to other ion channels, these data nevertheless underscore the necessity to break down ion channel expression at the subcompartment level to obtain a comprehensive view of their influence on neuronal excitability and how binge drinking might affect dendritic and somatic excitability independently. Finally, it is important to underscore the idea that an ion channel insensitivity to acute EtOH does not necessarily disqualify it as a potentially important alcohol target. Indeed, as noted above, acute EtOH fails to alter I_A_ (Kv4) potassium channel properties. Yet, prolonged exposure to the drug reduces expression of I_A_ channels (Mulholland et al., 2015), an effect associated with increased backpropagating action potential-evoked Ca^2+^ transients in the distal apical dendrites of CA1 pyramidal neurons that may profoundly alter synaptic integration.

## 4. Binge drinking modulation of synaptic transmission and plasticity

In addition to modulating intrinsic neuronal excitability, binge drinking alters the strength of synaptic transmission mediated by a number of neurotransmitters. Glutamate- and GABA-mediated synaptic transmission have been the major focus of past research owing to their central role in the nervous system as the primary excitatory and inhibitory neurotransmitters, respectively, as well as to their sensitivity to acute EtOH being an area beneficiated by the impulse in transgenic and gene “knockout” development (Hoffman et al., 2001). Unlike most voltage-gated K+ channels, both glutamate and GABA receptors are highly sensitive to low acute EtOH concentrations. While physiologically relevant concentrations of acute EtOH (i.e., 5–50 mM) inhibited NMDA receptor-mediated transmission in the hippocampus (Lovinger et al., 1990), the PFC (Weitlauf and Woodward, 2008), the amygdala (Roberto et al., 2004), and the NAc (Nie et al., 1994), they consistently enhanced GABA_A_ receptor in expression systems (Harris et al., 1995; Dildy-Mayfield et al., 1996), neuronal cell culture, and in fresh slices of tissue from the cortex and the amygdala (Celentano et al., 1988; Deitrich et al., 1989; Aguayo and Pancetti, 1994; Mehta and Ticku, 1994) as well as the NAc (Nie et al., 2000). As with BK channels, interactions between EtOH and NMDA/GABA_A_ receptors (Blednov et al., 2011; den Hartog et al., 2013) likely result from the effect of EtOH on the channel itself since it is limited by the size of its carboxyl chain (Lobo and Harris, 2008).

### 4.1. Binge drinking and glutamate-mediated synaptic transmission

Receptors for the amino acid L-glutamate that contribute to excitatory synaptic transmission are expressed throughout the brain and spinal cord. Beginning in the late 1970s, glutamate receptors in the vertebrate central nervous system (CNS) were classified into three families on the basis of pharmacological tools such as the agonists α-amino-3-hydroxy-methylisoxazolepropionic acid (AMPA), kainic acid, and N-methyl-D-aspartic acid (NMDA), and the antagonists such as 2- amino-5-phosphonopentanoic acid (AP5), also referred to as APV, and 6-cyano-7-nitroquinoxaline-2,3-dione (CNQX; Watkins and Evans, 1981). Subsequently, it was shown that while the majority of the first-recognized glutamate receptors were ligand-gated ion channels, a large number of G protein-coupled (i.e., metabotropic) glutamate receptors are also expressed throughout the CNS (Nakanishi, 1992). For the purpose of this review, we will limit the discussion to AMPA and NMDA receptors because of their central role in both synaptic transmission and plasticity. Further description of the glutamate transmission in the CNS can be found in Zhou and Danbolt (2014).

NAc can be divided into two anatomically and functionally distinct subregions: The sell and the core. While the core is involved in motor functions related to reward among other behaviors, the shell is involved in the cognitive processing of reward (Malenka et al., 2009). In the core, but not the shell, two subdivisions of the nucleus accumbens that present different functional and anatomical properties (Salgado and Kaplitt, 2015), binge drinking increases the expression of the gene encoding the NMDA receptors NR2B subunit but not the NR2A subunit, two NMDA receptor auxiliary subunits that are components of the heteromultimeric NMDA receptors. In primary cultured cortical neurons, the surface expression of both NR1 and NR2B subunits increased after CIE treatment (Qiang et al., 2007), a finding that mirrors a similar increase of NR2A and NR2B membrane expression in CA1 hippocampal neurons following 7 days of withdrawal from CIE treatment, a phenomenon that was accompanied by a significant enhancement of NMDA receptor-mediated synaptic response (Nelson et al., 2005). However, in the orbitofrontal cortex, CIE led to an increase of the AMPA/NMDA ratio in part through a decrease of NMDA NR2B subunit expression (Nimitvilai et al., 2016). Interestingly, changes of NMDA receptor subunit expression do not appear to occur in all addiction-related brain regions. Thus, in the CeA, a liquid diet treatment showed no effects on NMDA NR1 and NR2B subunit expression (Lack et al., 2005). Although the mechanisms leading to changes in genes expression remain poorly understood, there is growing evidence that histone demethylation at H3K9 and H3K36 in PFC and hippocampus may be implicated (Qiang et al., 2014; Finegersh et al., 2015; Simon-O’Brien et al., 2015; Sakharkar et al., 2016; Ponomarev et al., 2017; Wolstenholme et al., 2017). Functionally, a similar treatment in adolescent rats leads to an increase of electrically-evoked NMDA currents in CA1 hippocampal pyramidal neurons (Swartzwelder et al., 2017). The opposite finding was reported in the medial PFC area where CIE reduced the amplitude of electrically evoked NMDA currents, possibly through the downregulation of the NMDA NR1 subunit expression (Holmes et al., 2012). As with EtOH’s effects on neuronal firing, these findings underscore again the complex, and sometime seemingly contradictory interactions between alcohol and NMDA receptors, likely reflecting brain regions, alcohol treatments, and neuronal subtypes heterogeneity.

Regarding AMPA receptors, intermittent access to alcohol in young rats induces an increase in basal glutamate in the NAc core that is accompanied by a decrease in the frequency of spontaneous AMPA events (Pati et al., 2016), confirming data from an earlier study in the same region (Griffin et al., 2014). Surprisingly, using western blotting, the authors also detected a marked increase in surface expression of the AMPA GluA1 subunit, a result that seems inconsistent with a lack of change in the amplitude of spontaneous EPSCs events A possible explanation for this apparent discrepancy between electrophysiological and western blotting data is that newly formed AMPA receptors were extrasynaptic. This could provide MSNs with a pool of readily available AMPA receptors for further rapid insertion of AMPA receptors at synapses. Combining new biosensors with high resolution microscopy may help resolve this issue (Choquet et al., 2021). At thalamic inputs to CeA neurons, CIE enhanced glutamate release probability, as shown by a reduction of paired-pulse facilitation (Christian et al., 2013). The relationship between AMPA and NMDA receptor subunits and binge drinking has been tested using a number of knock out mouse models. Surprisingly, in a 2-bottle choice drinking model, Cowen et al. (2003) found no significant differences between the wild-type and AMPA GluR1 KO mice in the acquisition of voluntary ethanol consumption (Cowen et al., 2003). A similar lack of EtOH consumption was reported in AMPA GluR3 KO mice compared to wild-type littermates (Sanchis-Segura et al., 2006). Regarding NMDA receptors, the absence of the NR2A subunit has no effect on EtOH consumption (Boyce-Rustay and Holmes, 2006). Taken together these data show that knocking down particular genes associated with AMPA and NMDA synaptic transmission has overall little impact on alcohol consumption, a somewhat surprising result considering the large body of research supporting a link between NMDA receptor pharmacology and alcohol dependence in humans (Krystal et al., 2003).

Although it might be tempting to dismiss the role of these ion channels in mediating alcohol effects on behavior, some explanation can be proposed to account for such apparent negative results (i) although some early studies showed no apparent compensatory mechanisms in the hippocampus in AMPA GluR1 using KO mice (Sakimura et al., 1995; Jia et al., 1996), it remains that this question has been examined in too few brain regions to draw broad conclusions as to the existence (or lack thereof) of such mechanisms in other regions associated with alcohol addiction, (ii) A more focused approach using conditional knockout techniques (e.g., the Cre-lox recombination system) might prove useful by enabling the removal of specific genes in specific neuronal populations.

### 4.2. Binge drinking and glutamate-mediated synaptic plasticity

There is agreement that at the heart of alcohol’s persistent effects on behavior lays its ability to modulate “Long-term potentiation” (LTP), and long-term depression or LTD (Lovinger et al., 2003; Zorumski et al., 2014). Although the tetanic stimulation originally described by Bliss and Lømo (1973) remains the stimulation paradigm of choice in the majority of brain regions studied, another induction protocol called spike-timing dependent plasticity (STDP) is now being used in the NAc (Ji and Martin, 2012) where classic tetanic stimulations fail to reliably induce LTP (Kombian and Malenka, 1994; Robbe et al., 2002; Schramm et al., 2002), and in the cortex (Kroener et al., 2012). STDP is based on the timing (typically within a critical time window of 10–20 ms) and the pairing order between pre-and postsynaptic action potentials at low frequency (i.e., ~ 1 Hz; Sjostrom et al., 2008). There are little doubts that the AMPA and NMDA receptors, which control the initial rise of intracellular calcium that triggers phosphorylation of calcium kinases, are essential for the induction of LTP. Regarding LTD, the situation is somewhat more complex. While NMDA receptors do play an important role in some brain regions and under some experimental conditions, they are not necessary in others. Our understanding of synaptic plasticity is further complicated in the NAc where several forms of LTD have been recorded. Those include the NMDA-, mGluR- (see for review Winder et al., 2002) and action potential-dependent LTDs (Ji and Martin, 2012), a situation that contrasts with that of the dorsal striatum where both LTD and LTP are NMDAR-dependent (Partridge et al., 2000).

Several groups have explored the changes in LTP and LTD in models of binge alcohol drinking (Roberto et al., 2002, 2003; Stephens et al., 2005; Bernier et al., 2011; Kroener et al., 2012; Agoglia et al., 2015; Risher et al., 2015; Gruol et al., 2021). Field potential recordings in CA1 hippocampus display a larger LTP at the lowest stimulus intensity tested in CIE Sprague–Dawley rats, a difference that was abolished when stronger intensities were tested (Risher et al., 2015). This result contrasts with earlier studies in the same brain region where the post-tetanic field potentials were totally inhibited by CIE treatment (Roberto et al., 2002), an effect the authors later attributed in part to inhibition of the MAP kinase pathway (Roberto et al., 2003), a family of intracellular signaling molecules that regulate synaptic plasticity and learning (Thomas and Huganir, 2004), and whose inhibition decreases binge drinking (Agoglia et al., 2015). These data were subsequently replicated, also in the CA1 hippocampus, in a different rat strain (Stephens et al., 2005). Interestingly, regarding LTD, only two consecutive binges of EtOH (9 h apart) were sufficient to fully inhibit it in the same region, an effect that was reversed 8 days following the last EtOH exposure (Silvestre de Ferron et al., 2015). In both the VTA and PFC, the amplitude of tetanic stimulation- and STDP-mediated LTPs is enhanced (Bernier et al., 2011; Kroener et al., 2012). Although how EtOH alters LTP is not fully understood, a key role for IL-6 has been proposed (Gruol et al., 2021). In the NAc, the situation is somewhat more complex owing to different stimulation protocols (i.e., tetanic vs. STDP) and to the existence of MSNs expressing either dopamine D1 or D2 receptors. While D1-MSNs project to the VTA and to some extend to the ventral pallidum, D2-MSNs project exclusively to the ventral pallidum (Gerfen, 1984; Kupchik et al., 2015). Importantly, D1-MSN activation is related to positive rewarding events, inducing persistent reinforcement, whereas D2-MSN signaling is thought to mediate aversion (Hikida et al., 2010; Lobo et al., 2010; Kravitz et al., 2012), although this dichotomy might be more complex than initially thought (Soares-Cunha et al., 2020). Yet, despite many limitations, some commonalities have emerged among all these studies. In the NAc shell, LTD was inhibited by CIE exposure in MSNs, an effect that dissipated 72 h following the last treatment (Jeanes et al., 2011). Following up on their initial observations, the same group demonstrated a remarkable complexity in basic synaptic plasticity properties in MSNs expressing D1 and D2 receptors, but also a remarkable divergence in their respective responses to binge drinking. Thus, in transgenic Drd1-eGFP naïve mice, they could evoke LTD in D1R- but not in putative D2R-expressing MSNs. Interestingly, four consecutive days of CIE treatment totally blocked LTD in the former, while they observed LTD in the latter MSNs population, an effect that took 2 weeks to reverse (Jeanes et al., 2014). Comparison between the shell and core subregions revealed that CIE-mediated inhibition of LTD in D1R-MSNs was circumscribed to the shell while the core region seemed unaffected by the treatment (Renteria et al., 2018). These data provide critical evidence that binge drinking differentially modulates synaptic plasticity in D1R- and D2R-expressing MSNs. The same group shows that CIE-induced escalation of EtOH consumption produces NMDAr-dependent LTD in D1R (Renteria et al., 2018). In the NAc core, the same STDP stimulation paradigm evoked both tLTP and tLTD. While LTP was conventionally NMDAR-dependent, tLTD was controlled entirely by backpropagating action potentials (Ji and Martin, 2012). In another study, our group showed that tLTD and tLTP were primarily found in D1R- and D2R-expressing MSNs, respectively (Ji et al., 2017b). Two weeks of binge drinking led plasticity to switch from tLTD to tLTP in D1R-expressing MSNs, mirroring the findings by the Morrisett group, while tLTP in D2R-expressing MSNs was only mildly and not significantly attenuated. Interestingly, alterations of plasticity by binge drinking were not accompanied by changes in AMPA or NMDA current properties at PFC, hippocampal or amygdala synapses; only significant changes of the AMPA/NMDA ratio, a widely accepted index of plasticity, were reported (Ji et al., 2017b).

As noted above, because spike-timing-dependent LTD is controlled by backpropagating action potentials, it is possible that the tLTD-to-tLTP switch reflects a weakening of these electrical events as they invade the dendritic tree retrogradely, a phenomenon that does not exclude insertion of AMPA receptors vs. changes in their subunit composition as seen in other brain regions. The strengthening of glutamate synaptic transmission in D1R-expressing MSNs seems to be supported by findings in the dorsal striatum of mice trained to consume alcohol using an intermittent-access two-bottle-choice drinking paradigm. In this study, Cheng et al. (2017) demonstrated a similar increase in the AMPA/NMDA ratio, increase found in D1R- but not in D2R-expressing MSNs. Using a chemogenetic approach, the study also reported that the excitation of D1R-expressing MSNs promoted alcohol drinking, establishing a strong correlation between the enhance excitation in these neurons and drinking. Much remains to be understood about the molecular underpinnings responsible for the differential effects of binge drinking on the glutamatergic synaptic transmission of striatal D1R- and D2R-expressing MSNs, and the increase of the AMPA/NMDA ratio. In light of NMDA receptor subunit expression sensitivity to alcohol, as seen in the previous section, binge drinking may trigger the expression of different NMDA receptor subunits with higher unitary conductance. Our group measured the deactivation rates of optogenetically-driven NMDA currents, a feature controlled by the receptor subunit composition (Monyer et al., 1992). Surprisingly, we showed that NMDA current decay kinetics remained unchanged after binge EtOH, seemingly ruling out this possibility (Ji et al., 2017b). Irrespective of the specifics of the alterations of the glutamate synaptic transmission in MSNs, binge drinking effects on NAc direct pathway (D1R) point to a remarkable degree of specificity that mirrors what has been reported with cocaine in the same brain region (Bock et al., 2013; Heinsbroek et al., 2017) and engages with the stablished dichotomy that shows that D1 is involved in reinforcement and reward and D2 has been associated with punishment and aversion (Soares-Cunha et al., 2016). It is likely that this specificity is functionally relevant as striatal D1R- and D2R-expressing MSNs project to different brain regions (Gerfen and Surmeier, 2011), even though new data are now challenging the strict anatomical segregation of these projections in mice NAc (Kupchik et al., 2015).

Importantly, alterations of the AMPA/NMDA synaptic transmission in the striatum may merely reflect alcohol-glutamate receptors interactions occurring simultaneously at multiple levels in the local circuitry. Indeed, glutamate synaptic transmission and plasticity in striatal MSNs are under the control of dopamine (Shen et al., 2008; Ji et al., 2017b), whose release is regulated in part by cholinergic interneurons (ACh INs). The putative role of these interneurons is particularly intriguing. Despite their rarity as they represent between 1 and 2% of the total neuronal population of the rat striatum (Lim et al., 2014), ACh INs are critical players in regulating the output of this region due to their direct connection to MSNs (Hsu et al., 1996; Galarraga et al., 1999; Shen et al., 2005), as well as through the control of dopamine (Threlfell et al., 2010; Cachope et al., 2012), GABA (Witten et al., 2010), and glutamate (Bonsi et al., 2011; de Kloet et al., 2015; Silberberg and Bolam, 2015) release from terminals that synapse on MSNs. Alterations of the properties of ACh INs have been associated with neurological disorders such as depression and emotional control (Warner-Schmidt et al., 2012; Atallah et al., 2014). They have also been implicated in a number of neurological disorders in task attention, memory, and aversive behavior (Aosaki et al., 1994; Ravel et al., 1999; Anagnostaras et al., 2003; Furey et al., 2008). With respect to drugs of reward and addiction, their spontaneous firing is influenced by event context and by cocaine reward-related cues *in vivo* preparations (Apicella et al., 1997; Williams and Adinoff, 2008; Witten et al., 2010; Schmidt et al., 2011; Tuesta et al., 2011; Atallah et al., 2014). There is also evidence that acetylcholine mediates EtOH’s effects by modulating the release of dopamine from terminals originating in the VTA (Adamantidis et al., 2011; Abrahao et al., 2012; Bahi and Dreyer, 2012; Engel and Jerlhag, 2014). Binge alcohol drinking in adolescents profoundly reduces both the expression of 11 of the 14 genes encoding nicotinic acetylcholine receptors (Colbert et al., 1997), and the density of ACh INs in the NAc of human alcoholics (Vetreno et al., 2014) as well as in an adolescent intermittent alcohol drinking model in rats (Hauser et al., 2019). Similarly, chronic alcohol consumption reduces the number of striatal cholinergic varicosities (Pereira et al., 2014). There is also evidence that ACh INs spontaneous firing is inhibited by EtOH (Blomeley et al., 2011). In a recent study, Kolpakova et al. (2022) detailed the complex cellular mechanisms underlying ACh INs-mediated inhibition of glutamate release in D1 and D2 MSNs. Thus, NAc ChIs decreased MSN synaptic excitability through different mechanisms in D1- vs. D2-MSNs. While decrease of ChI-mediated sEPSCs frequency in D1-MSNs was mediated by dopamine, the same effect in D2-MSNs resulted from a direct control of glutamate release by ChIs. Interestingly, after 2 weeks of binge alcohol drinking, optogenetic stimulation of ChIs enhanced glutamate release in D1-MSNs, while its effect on D2-MSNs remained unchanged (Kolpakova et al., 2022). Taken together, these studies suggest that binge drinking may initiate a carefully orchestrated cascade of events, starting with ACh INs through their control of dopamine release, that ultimately favors synaptic transmission and plasticity of MSNs belonging to the direct pathway. Also, restoring the balance between the NAc direct and indirect pathways in favor of the latter, possibly through manipulations of ACh INs excitability, may help protect individuals against the long-term consequences of alcohol consumption.

### 4.3. Synaptic gating as a model of synaptic adaptation to binge alcohol drinking

The nucleus accumbens receives glutamatergic afferents primarily from the prefrontal cortex, hippocampus, basolateral amygdala (BLA), and hippocampus (Ikemoto, 2007; Humphries and Prescott, 2010; Li et al., 2018), allowing integration of emotional, contextual, and cognitive information. There is evidence that these inputs do not behave independently of each other. Thus, early *in vivo* recordings by O’Donnell and Grace (1995) revealed that hippocampal inputs facilitate the transmission of information originating in the prefrontal cortex in NAc MSNs, a mechanism called synaptic gating (O’Donnell and Grace, 1995; Katz, 2003). In 2005, based in part on earlier observations in human subjects (Bechara et al., 1995), Bechara proposed that the loss of control over alcohol consumption in adolescents was be caused by an imbalance between the reflective and impulsive systems that are, respectively, associated with the prefrontal cortex (PFC) and the basolateral amygdala (BLA), respectively (Bechara, 2005). While the PFC is responsible for planning, evaluating long-term consequences and is instrumental in retrieving drug-associated memories (Dalley et al., 2004; Zhang et al., 2019), the BLA encodes emotions that shape impulsive behavior and the response to associative learning (Gallagher and Chiba, 1996; Cardinal et al., 2002; Lalumiere, 2014). More specifically, Bechara hypothesized that the impulsive system could override the reflective system upon repeated drugs of abuse consumption. To identify the molecular basis of a putative disruption of synaptic integration between cortical and amygdala inputs, Kolpakova et al. (2021) used a double optogenetics approach (i.e., Channelrhodopsin and ChrimsonR) to independently stimulate the PFC and BLA afferents, respectively, to elucidate how executive and emotional information is processed by MSNs in alcohol-naïve and binge alcohol drinking mice (Kolpakova et al., 2021). This approach revealed that PFC and BLA inputs synapse onto the same MSNs where they reciprocally inhibit each other presynaptically in a strict time-dependent manner. In alcohol-naïve mice, this temporal gating of BLA-inputs by PFC afferents is stronger than the reverse, revealing that MSNs prioritize high-order executive processes information from the PFC. Importantly, binge alcohol drinking alters this reciprocal inhibition by unilaterally strengthening BLA inhibition of PFC inputs. In line with this observation, we demonstrate that *in vivo* optogenetic stimulation of the BLA, but not PFC, blocks binge alcohol drinking escalation in mice (Kolpakova et al., 2021). Overall, this study identified a new mechanism through which NAc MSNs integrate executive and emotional information and showed that this integration is dysregulated during binge alcohol drinking. It also highlights the idea to fully understand the effects of alcohol on synaptic transmission and neuronal excitability in the NAc and elsewhere it is important to consider the network in its globality and diversity, and the dynamic interplay between various inputs (Kolpakova et al., 2021).

### 4.4. CIE and GABA- and glycine-mediated synaptic transmission

GABA_A_ receptors are formed by the association of 5 subunits (α1-6, β1-3, γ1-3, δ1, ε, Θ, and π) asymmetrically arranged around the central chloride anion conduction pore. Although their stoichiometry and composition vary with cell types and brain regions, most neurons express a combination of αβγ subunits (Sigel and Steinmann, 2012). Of all these subunits, the α subunits seems to be particularly sensitive to binge drinking.

Thus, the examination of subunit expression in the hippocampus revealed a decrease of mRNA encoding the α1 and an increase in α4 subunit mRNA expression in CIE rats (Cagetti et al., 2003). In the dentate gyrus, protein levels for the α4-GABA_A_ receptor subunits were significantly reduced, but mRNA levels were increased, 26 days after the last intermittent alcohol exposure in mice (Centanni et al., 2014). Interestingly, in the same publication, Cagetti et al. (2003) found no effect on expression of any of these subunits following CIE exposure during adulthood, underscoring the idea of adolescence as a time window particularly sensitive to the effect of EtOH. While a two-day withdrawal following CIE treatment leads to a significant increase in the α4 subunit mRNA levels in the dentate gyrus, the CA3, and the CA1 regions, no significant change in the α5 subunit expression was observed in the same regions. There is also evidence of a reorganization of the α4 synaptic and extrasynaptic GABA_A_ receptor subunit in CA1 pyramidal neurons (Liang et al., 2006). A similar reorganization of GABA_A_ receptor subunit composition in the hippocampus of CIE animals appears to be supported by mIPSCs with markedly different activation/deactivation kinetics, an effect that was associated with upregulation of the α2, and not the α4, subunit, an effect the authors associated with the anxiolytic response to EtOH exposure (Lindemeyer et al., 2017). Interestingly, a point mutation that renders the α2 subunit insensitive to benzodiazepine blunted alcohol intake in mice with an intermittent access to EtOH, suggesting that neurosteroid action on α2-containing receptors may be necessary for escalation of chronic EtOH intake (Newman et al., 2016). The role of the α2 subunit appears to be supported by data showing that a point mutation from histidine at 270 to alanine decreased alcohol consumed and reduced preference for ethanol (Blednov et al., 2011). Also in the hippocampal CA1 pyramidal neurons, intermittent ethanol exposure modulated GABA_A_R δ but not α4 subunit expression (Follesa et al., 2015). Regarding the δ-GABA_A_ receptors, a significant reduction in its protein levels was observed in the dentate gyrus, in the absence of any changes in mRNA levels, at 48 h and 26 days after the last ethanol CIE exposure (Centanni et al., 2014). Possibly related to changes in subunit expression, in hippocampal dentate granule cells, alcohol exposure during adolescence decreased the tonic noise driven by extrasynaptic GABA_A_ receptors, in CIE animals compared to untreated animals, suggesting a weaker baseline inhibition (Fleming et al., 2012). In CIE rats, GABA_A_ receptor-mediated synaptic transmission was depressed in CA1 hippocampus as suggested by a decrease in agonist-evoked ^36^Cl^−^ efflux, of the paired-pulse inhibition in the CA1 area, and alterations of GABA_A_ receptor subunit expression. Taken together, these studies seem to point to the hippocampal α2 and α4 subunits as being particularly sensitive to binge drinking.

In other brain regions, similar adaptations of GABA receptor subunit expression were observed, with again the α subunits playing a central role. Thus, in rat cerebellum, an increase of GABA_A_ α6 subunit expression was detected following CIE treatment (Petrie et al., 2001). In the PFC, CIE decreased the levels of α1 and α2 subunits, and increased the level of α4 (Sheela Rani and Ticku, 2006), while it reduced the amplitude of tonic GABA currents in layer V pyramidal neurons, perhaps reflecting an attenuation of currents mediated by δ-subunit containing receptors (Centanni et al., 2017). Finally, a recent study shows that in these regions, withdrawal impairs α1subunit affecting synaptic neurotransmission (Hughes et al., 2019). In the NAc, CIE decreased the frequency of fast-rising miniature IPSCs (Liang et al., 2014a), mirroring reports in dorsal striatum MSNs (Wilcox et al., 2014), an effect that the authors interpreted as consistent with a possible decrease in somatic GABAergic synapses in MSNs from CIE rats. In a separate study, the same group reported that dopamine, at concentrations consistent with those measured *in vivo* (0.01–1 μM), modulated extrasynaptic GABA_A_ receptors of NAc MSNs, without affecting the postsynaptic kinetics of miniature inhibitory postsynaptic currents (mIPSCs), highlighting the differential sensitivity of synaptic and extrasynaptic GABA receptors to CIE treatment (Liang et al., 2014b). Interestingly, RNA sequencing demonstrated that the expression of most (6 of 8) GABA_A_ receptor subunit genes decreased in the periacqueductal gray (McClintick et al., 2016) and increased in the dorsal raphe (McClintick et al., 2015) following CIE treatment. Because these brain regions are not believed to play a direct role in addiction but rather are central in processing pain and anxiety, it seems somewhat surprising to see GABA_A_ receptor subunit expression change in response to intermittent EtOH exposure. However, this effect may be related to finding by Fu et al. (2015) who showed that CIE induces hyperalgesia in rats and to the well-known anxiogenic effects of repeated EtOH consumption associated with withdrawal (Hamilton et al., 2013). Finally, in the CeA region, CIE effects on tonic GABA-mediated synaptic transmission is complex due in part to the presence of different neuronal populations (i.e., low-threshold bursting [LTB], regular spiking and late spiking neurons), a mosaic further complicated by the expression (or not) of the corticotropin releasing factor receptors (CRF1). While the frequency of spontaneous events (i.e., phasic GABA response) was markedly reduced in CeA neurons expressing the CRF1 receptor, it was enhanced in CRF1-negative late spiking cells (Herman et al., 2016). Additionally, the study reported a loss of the tonic GABA current in CRF1 but not in CRF1-negative neurons, an effect that persisted into withdrawal.

These data highlight two key facts. First, the expression of GABAR α subunits seems to be particularly sensitive to repeated alcohol exposure. Second, they underscore the idea that GABA-EtOH interactions are complex and cannot be strictly described in terms of inhibitory or excitatory effects. These seemingly disparate outcomes may reflect the unique expression pattern of the GABA receptor subunits in different neuronal populations and their response to binge drinking. Equally important is that the overall effects of CIE on neuronal excitability through GABA_A_ synaptic transmission depend on the specific electrical characteristics of the neurons studied and particularly on their resting membrane potentials (RMPs). Thus, considering that the reversal potential for chloride (E_Cl_^−^) typically ranges between −70 and −75 mV in nerve cells (Deisz and Prince, 1989), neurons presenting more depolarized RMPs will display increased excitability upon a decrease of inhibitory activity, due to weakening of the ability of GABA_A_ receptors to prevent the cells from further depolarizing. In contrast, in neurons whose RMP is much more hyperpolarized (i.e., ~ − 85 mV) such as NAc MSNs, GABA_A_ receptors likely mediate depolarization at rest. As such, a weakening of the GABA transmission will further dampen MSNs neuronal excitability. Regarding CIE effects on GABA tonic currents, in neurons with resting potentials close to E_Cl_^−^, this tonic current is likely to shunt the membrane, which would primarily negatively impact neuronal cable properties and the propagation of synaptic events from their point of inception in spines to the soma by attenuating the amplitude of these events and slowing their kinetics. Therefore, associating changes of GABA synaptic transmission with an overall inhibitory or excitatory effect should be carefully weighted in light of the basic electrical properties of the neurons considered.

Less is known about interactions between alcohol and the GABA_B_ receptor, a metabotropic receptor that inhibits neuronal excitability through its action on g-protein-coupled inward rectifying potassium (GIRK) channels (Padgett and Slesinger, 2010). In BLA neurons, GABA_B_ receptors appear to be responsible for tolerance to acute ethanol-mediated increase in the frequency of spontaneous GABAergic synaptic currents (Zhu and Lovinger, 2006). Interestingly, in the hippocampus, inhibition of GABA_B_ receptor function enhances ethanol-mediated potentiation of distal GABA_A_ IPSCs (Proctor et al., 2006), bolstering the idea that they may counter the effects of EtOH on GABA_A_ receptors. In a two-bottle choice alcohol drinking model, Herman et al. (2015) showed that constitutive deletion of GIRK_3_, one of the three GIRK subunits, selectively increased ethanol binge-like drinking. Additionally, they reported that GIRK_3_ is responsible for EtOH-mediated increase of VTA DA neurons firing and EtOH-mediated DA release in the NAc.

Glycine, alongside GABA, is the other fast inhibitory neurotransmitter in the central nervous system. Like GABA receptors, activation of glycine receptors opens an anionic conductance that hyperpolarizes the membrane potential. Although it was initially believed that glycine receptors were almost exclusively found in the spinal cord and brainstem of adult rats (Rajendra et al., 1997), their expression was subsequently reported in forebrain structures associated with the addiction neurocircuitry (Yoon et al., 1998; McCool and Botting, 2000; McCool and Farroni, 2001; Martin and Siggins, 2002; Mori et al., 2002). As with GABA receptors, acute EtOH generally enhances glycine currents in a number of preparations (Celentano et al., 1988; Aguayo and Pancetti, 1994; Mascia et al., 1996; Ye et al., 2001) even though inhibition of glycine currents was also reported in the ventral tegmental area (Tao and Ye, 2002). Interestingly, in the lateral orbital frontal cortex, acute EtOH enhances glycine currents without affecting GABA currents (Badanich et al., 2013). Unfortunately, there is currently little information as to how these receptors adapt to binge drinking. On the model of GABA receptors and other ionotropic receptors, the GlyR is a pentameric receptor constituted as either α-homomers or α-β heteromers. In the amygdala and NAc, GlyR α2 and α3 subunits show equal or greater expression compared with α1 (Jonsson et al., 2009; Delaney et al., 2010). In light of the sensitivity of the expression of the various subunits forming GABA and AMPA/NMDARs, it is tempting to speculate that GlyRs may similarly respond to binge drinking by altering their subunit composition, an adaptation that would not only influence their intrinsic channel properties (Grudzinska et al., 2005) but also their location (Laube et al., 2002). A study by McClintick et al. (2016) showed a reduced expression of 4 glycine receptor-related genes in the periacqueductal gray of DID rats. Recently the field has been expanded to acknowledge the role that other glycine receptor agonists, such as Taurine, can play in alcohol dependence, in this sense has been demonstrated that alcohol dependence disrupts the taurine-mediated inhibition of the GABAergic tone in the amygdala (Kirson et al., 2020).

Behaviorally, microinjections of glycine into the VTA decreased EtOH intake, but not sucrose or water, in rats chronically exposed to ethanol under the intermittent-access protocol (Li et al., 2012). Regarding GABA receptors, their role has been examined using both the two-bottle choice and the DID models in knockout mouse. While all GABAR subunits do not have the same influence on binge drinking, those that do alter the behavior consistently decrease drinking. Thus, alcohol consumption of α1, α5 and δ knockout mice was lower than that of wild-type littermates (Mihalek et al., 2001; Blednov et al., 2003; Boehm et al., 2004; June et al., 2007). In contrast, α2 and β2 knockout mice had no effects on alcohol consumption (Blednov et al., 2003; Boehm et al., 2004). Although limited in scope, these studies appear to indicate that alterations of GABA and Glycine-mediated inhibitory synaptic transmission attenuate alcohol drinking.

## 5. What is the role of ion channels on neuronal excitability in binge alcohol drinking animals?

Data presented here offer a strong rationale for some voltage- and ligand-gated ion channels as potential direct candidates that mediate the effects of binge alcohol drinking on neuronal excitability and communication. Indeed, for good reasons, glutamate has long held a central role in various models of addiction (Berke and Hyman, 2000; Kauer and Malenka, 2007; Kalivas, 2009; Kalivas et al., 2009). However, to fully appreciate the role of glutamate in binge alcohol drinking and how precisely it exerts its effects, it is critical to understand not only how individually it affects neuronal excitability and how AMPA/NMDA receptors properties are influenced by alcohol, but also how they work in the broader context of the constantly changing and dynamic neuronal excitability. As already alluded to in this review, voltage- and ligand-gated channels do not work separately and independently. For example, there is a wealth of evidence that membrane depolarization following the release of glutamate recruits a number of voltage-gated calcium and potassium channels that contribute to modulating the kinetics of synaptic potentials (Cai et al., 2004; Kim et al., 2007; Lin et al., 2008) and by extension how synaptic events temporally and spatially aggregate to trigger action potentials and how they may in some situations modulate neurotransmitter release (Debanne et al., 2013). This is probably a phenomenon more exacerbated in dendritic spines than in synapses on the dendritic shaft and cell body since spines are a fairly well isolated electrical compartment. Thus, when considering the modulation of LTP by binge drinking, one may want to go beyond the generally accepted and critical role of AMPA and NMDA receptors to include a direct participation of voltage-gated ion channels. For example, Ia, SK and BK channels are widely expressed in both dendritic spines where they control glutamate-mediated depolarization and calcium influx (Isaacson and Murphy, 2001; Faber et al., 2005; Wang et al., 2014). By altering their subunit composition or by undergoing endocytosis in response to binge drinking, they may contribute to changing the long-lasting strength of synaptic transmission independently of the effects of alcohol on AMPA/NMDA receptors. Thus, in the NAc, binge drinking promotes LTP in D1R-MSNs, an effect that is not accompanied by a corresponding increase of AMPA/NMDA ratio, a classic measure of AMPA receptor insertion, at hippocampal glutamatergic synaptic inputs compared to cortical and amygdala synapses (Ji et al., 2017b). This suggests that the mechanisms underlying synaptic plasticity may differ in the same neuronal population based on the origin of the afferents. While interacting with ligand-gated channels in dendrites, the same ion channels will concomitantly shape action potentials generated at their point of inception in the initial segment near the soma. By the same token, when expressed in the membrane of the dendritic shaft, they will not only modulate the strength of back-propagating action potentials but also the degree of filtering to which synaptic events reaching the soma are subjected based on the cable theory proposed by W. Rall more than 50 years ago (RALL, 1959). This shows that to fully capture the complexity of the contribution of ion channels to neuronal excitability necessitates integration of various dimensions of all ion channels in a unifying model, a goal that we have not reached yet, in part due to the challenges of probing hard-to-reach neuronal compartments such as dendritic spines.

## 6. Future perspectives

The present survey of the literature is a testimony to the progress accomplished over the recent decades toward understanding how neurons of the central nervous system adapt to repeated alcohol consumption. To some measure, the origin of each advance can be traced back to specific technological breakthroughs. Thus, following the widespread adoption of the voltage-clamp technique that enabled whole-cell and single-channel recordings of specific ion channels in the 80s, the 90s capitalized on the advancement of cloning techniques to identify the many subunits of a host of ligand- and voltage-gated ion channels, to determine their influence on biological and pharmacological properties of these channels, and to establish their influence on interactions with alcohol. The turn of the century witnessed dramatic progress in genome wide sequencing analysis that led to a better appreciation of the expression patterns of these targets as well as many others, in binge drinking animals, and the regulation of their expression by non-coding RNA and epigenetic mechanisms. More recently, opto- and chemogenetic approaches have been instrumental in helping to disentangle the complex neuronal circuitry by enabling the targeting of specific pathways or neuronal populations, a goal that was for the most part unattainable with more conventional pharmacological and electrical approaches. Yet, despite this wealth of information, it remains difficult to ascribe EtOH a simple excitatory or inhibitory value. As highlighted here, EtOH’s influence on ion channel physiology and more broadly on neuronal excitability depends on a number of variables such as the ion channel subunit composition, their sensitivity to the drug and their site of expression (e.g., soma vs. dendrites). Also, when studying the effects of binge drinking on a particular ion channel, we tend to draw conclusions through the prism of this channel while ignoring all other interactions. All these factors combined often lead to a truncated interpretation of the influence of binge drinking on brain function. Interestingly, during the last years a vast body of evidence regarding the role that the epigenetic changes plays in alcohol consumption is growing and shows clearly that the adaptations produced by the epigenetic landscape and their interlinked pathways plays a key role in the development of AUD (see for review Egervari et al., 2021). Finally, a emboldened body of evidence shows that the immune system and stress can also modulate the effect of alcohol in the brain in different stages of consumption (i. e. binge drinking, tolerance, dependence) being a promising area of study (de Guglielmo et al., 2019; Blednov et al., 2021).

The contribution of these bottom-up approaches do not mask the fact that a broad narrative of the effects of binge drinking on brain function remains elusive, and poses the question of the best way forward in decades to come. It could be argued that a renewed focus on top-down approaches, typically represented by *in vivo* simultaneous recordings of multiple neurons, may prove beneficial for the field assuming that recent technological progress are harnessed. As successful as such an approach has proved in the past in providing a general sense of the overall neuronal excitability in any given brain region, it has been limited in its ability to concomitantly pinpoint specific neurons or their inputs. Fortunately, such limitations are fading as new techniques become available. One exciting recent development is the miniaturized fluorescence microscopy system that simultaneously monitor calcium transients (a proxy for neuronal excitability) in a large number of visually identified individual neurons in freely moving mice and rats (Kitamura et al., 2017). Through this approach, it is now possible to study the concept of the engram (i.e., a collection of neurons simultaneously activated) and to identify the location and physical basis of “memory traces” left in the brain by repeated binge drinking. Although miniaturized fluorescence microscopy remains the purview of few laboratories due to its cost and complexity, its dissemination in years to come will undoubtedly be instrumental in advancing the field of alcohol research.

Interestingly, a number of laboratories are now leveraging the power of sophisticated transgenic mice models (e.g., fos/arc-TRAP2, TetTag, and fos-^TVA^ mice) initially developed to identify and characterize neurons encoding fear retrieval memories (Liu et al., 2012; Sakurai et al., 2016; DeNardo et al., 2019), with various approaches like viral delivery technique, opto- and chemogenetics to establish a causal relationship between engram excitability and alcohol consumption and to elucidate what makes these neurons unique at the molecular (i.e., transcript profile) and functional (electrophysiological properties) levels. This approach may offer a unique opportunity to develop therapeutics that would selectively target neurons recruited by alcohol, such as the neuronal ensemble described in the amygdala that plays a key role in alcohol dependence (de Guglielmo et al., 2016) potentially resulting in higher efficacy and fewer side effects unlike what is reported with currently available drugs (Litten et al., 2016).

## Author contributions

GM wrote the manuscript with inputs from PG-G and TL. All authors contributed to the article and approved the submitted version.

## Funding

This work was supported by the National Institute on Alcohol Abuse and Alcoholism, grant nos. AA027807 and AA020501.

## Conflict of interest

The authors declare that the research was conducted in the absence of any commercial or financial relationships that could be construed as a potential conflict of interest.

## Publisher’s note

All claims expressed in this article are solely those of the authors and do not necessarily represent those of their affiliated organizations, or those of the publisher, the editors and the reviewers. Any product that may be evaluated in this article, or claim that may be made by its manufacturer, is not guaranteed or endorsed by the publisher.
